# Efficient electrocatalytic water splitting performance of an SrTiO_3_/g-C_3_N_4_ composite for hydrogen evolution in an acidic medium

**DOI:** 10.1039/d6ra01249c

**Published:** 2026-04-22

**Authors:** Leena Baskar, Divyadharshini Satheesh, Paul Joseph Daniel, Vijayarangamuthu Kalimuthu, Kovendhan Manavalan

**Affiliations:** a Department of Physics and Nanotechnology, SRM Institute of Science and Technology Kattankulathur Chengalpattu-603203 Tamil Nadu India mkovendhan@gmail.com; b Department of Physics, National Institute of Technology-Warangal Telangana State-506004 India; c Department of Physics, Pondicherry University Kalapet Pondicherry-605014 India

## Abstract

Water splitting is a research field that possesses a high scope for the generation of non-polluting and sustainable energy. In this study, an SrTiO_3_/g-C_3_N_4_ heterostructure composite is fabricated to boost the catalytic efficacy for the HER applications. The SrTiO_3_/g-C_3_N_4_ heterostructure composite was prepared *via* a hydrothermal technique by combining strontium titanate (SrTiO_3_) nanopowders with 2D graphitic carbon nitride (g-C_3_N_4_). The formation of the composite heterostructure between SrTiO_3_ and g-C_3_N_4_ was confirmed using XRD, SEM, and UV and Raman spectroscopies. The synergy between SrTiO_3_ and g-C_3_N_4_ played a key role in enhancing the catalytic efficacy. By combining the unique aspects of SrTiO_3_ and g-C_3_N_4_, the fabricated heterostructure composite achieved a low overpotential of 321 mV and a Tafel slope of 192 mV dec^−1^, indicating improved HER activity and thereby making it an effective catalyst for the hydrogen evolution reaction. The stability of the heterostructure composite was explored for prolonged reaction periods, and by using this composite, hydrogen generation was realized in a sustainable and efficient manner. This strategy is a promising approach to clean and renewable hydrogen generation.

## Abstract 2

Water splitting is a research field that possesses a high scope for the generation of non-polluting and sustainable energy.

## Graphical abstract

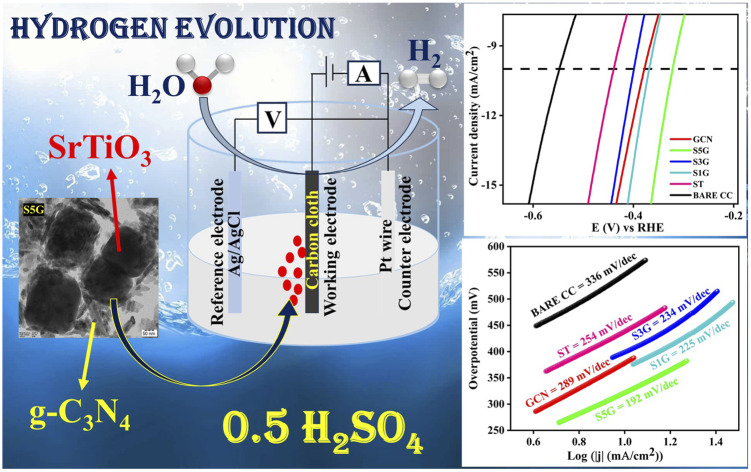

## 1. Introduction

The search for sustainable energy options is accelerated by the growing demand for clean and renewable energy on a global scale. Due to its enormous energy density and zero carbon emissions when in use, hydrogen (H_2_) has become one of the most promising sources of energy.^1^ One of the most effective and environmentally friendly routes for producing hydrogen is electrochemical water splitting, which is composed of two important half-reactions: the hydrogen evolution reaction (HER) at the cathode and the oxygen evolution reaction (OER) at the anode.^2^ The hydrogen evolution reaction (HER) is crucial for producing hydrogen gas, and it occurs by reducing protons in acidic conditions or by reducing water molecules in alkaline conditions, ultimately liberating hydrogen.^3,4^ Platinum (Pt)-based catalysts have shown enhanced performance for the HER because of their low overpotentials and high exchange current densities.^5,6^ Nevertheless, the high cost and scarcity of platinum hinder their scalability for real-time applications. This has led to intensive research into earth-abundant, low-cost alternative materials, such as transition metal compounds, metal-free catalysts, and heterostructured electrocatalysts.^7^

Strontium titanate (SrTiO_3_) has an ideal cubic perovskite-like unit cell of the ABO_3_ oxide system, wherein the Sr^2+^ and Ti^4+^ ions constitute the A and B sites, respectively. Nevertheless, an ideal perovskite-like STO shows lattice distortion at different levels, which determines the crystal field and, eventually, the dipole and electronic band structures. This lattice distortion also influences the photoinduced charge carrier aspects of STO, which is crucial for photocatalytic steps like excitation, transfer, and redox reactions.^8,9^ Perovskite-type strontium titanate (SrTiO_3_)^10^ has attracted attention in photocatalysis and electrocatalysis due to its chemical stability, tunable electronic structure, and good conductivity. SrTiO_3_ exhibits suitable conduction and valence band positions that can support the HER under acidic conditions. However, its practical application is often limited by its wide bandgap (∼3.61 eV), which restricts visible light absorption, and by its relatively low intrinsic catalytic activity. To overcome these limitations, graphitic carbon nitride (g-C_3_N_4_) has been explored as a supporting material.^11–13^ g-C_3_N_4_ is a metal-free polymeric semiconductor with a narrower bandgap (∼2.86 eV), enabling better visible-light absorption. It also possesses a layered structure, good chemical stability, and potential active sites for the HER. Often, g-C_3_N_4_ alone suffers from fast electron–hole recombination and limited charge mobility.^14,15^

By combining SrTiO_3_ with g-C_3_N_4_, a heterojunction composite can be formed, which synergistically enhances the overall electrocatalytic performance.^16,17^ The characteristics of the heterostructure composite facilitate improved charge separation, extend the light absorption range, and increase the number of active sites. The interaction of SrTiO_3_ with g-C_3_N_4_ at the interface helps in suppressing recombination losses and boosts catalytic efficiency under electrochemical conditions.^18,19^ In this context, the SrTiO_3_/g-C_3_N_4_ composite presents a promising strategy for developing efficient and sustainable HER electrocatalysts. This work aims to investigate the synthesis, characterization, and HER performance of the SrTiO_3_/g-C_3_N_4_ composite and to elucidate the synergistic mechanisms contributing to its enhanced catalytic behavior.^20,21^

## 2. Materials and experimental methods

Titanium butoxide (Ti(OBu)_4_), strontium nitrate (Sr(NO_3_)_2_), and sodium hydroxide (NaOH) were procured from Sisco Research Laboratories Pvt. Ltd, and melamine (99%) was obtained from Sigma-Aldrich. All chemicals and ethylene glycol solvent were used as procured without any further purification.

### 2.1. Synthesis of SrTiO_3_

5 mmol of Ti(OBu)_4_ was dissolved in 25 mL of ethylene glycol by stirring. Following the addition of 5 mL of 5 M NaOH solution, 10 mL of 0.5 M Sr(NO_3_)_2_ solution was added to the solution. After mixing for 10 min, the contents were transferred into a 50 mL Teflon-lined stainless steel autoclave and treated at 180 °C for 24 h. The resultant solid particles were collected by centrifuging with deionized water and ethanol, respectively. The solution is then dried at 70 °C for 12 h, and a white colour precipitate confirmed the formation SrTiO_3_.^22^

### 2.2. Preparation of the SrTiO_3_/g-C_3_N_4_ composite

g-C_3_N_4_ was prepared by treating melamine at 550 °C in a box furnace for 4 h, and a yellow-colored powder was obtained. The preparation of the SrTiO_3_/g-C_3_N_4_ heterostructure involved combining the prepared SrTiO_3_ nanoparticles and g-C_3_N_4_ sheets by ultrasonication.^23^ Heterostructures with different compositions of g-C_3_N_4_ (1, 3 and 5%) were dispersed in water, followed by the addition of SrTiO_3_ powders and then ultrasonicated for 1 h and mixed for 30 min. Finally, the mixture was treated at 80 °C for 12 h to obtain 1%, 3% and 5% g-C_3_N_4_-incorporated samples. The final product was obtained for different concentrations and are named as ST, GCN, S1G, S3G, and S5G.^24^

### 2.3. Mechanism of the HER under acidic conditions

The hydrogen evolution reaction (HER) is a process that leads to the generation of hydrogen gas at the surface of the working electrode. The chemical pathway varies depending on whether the electrolyte is acidic or alkaline. Under acidic conditions, the HER mechanism begins with the Volmer step (eqn (1)), which involves the electrochemical discharge of a proton. This is followed by either the Tafel reaction (eqn (2)), the Heyrovsky reaction (eqn (3)) or a combination of both.^25^

1 H_3_O^+^ + e^−^ + M → H_ads_ + H_2_O (Volmer)

2 H_ads_ + H_ads_ → H_2_ (Tafel)

3 H_ads_ + H_3_O^+^ + e^−^ → H_2_ + H_2_O (Heyrovsky)

In the Volmer–Heyrovsky mechanism, the initial step corresponds to proton adsorption (eqn (1)), followed by hydrogen evolution *via* the Heyrovsky step (eqn (3)). Here, an adsorbed hydrogen atom (H*) reacts with a proton from the acidic medium and an electron from the surface of the electrode to form molecular hydrogen (H_2_) and water. This pathway typically dominates when the surface coverage of adsorbed hydrogen is low, allowing for efficient electron transfer and product formation.

The Tafel curve, derived from the Tafel relation:

4 *η* = *a* + *b* log(*j*)

illustrates a linear behaviour between the overpotential (*η*), Tafel slope (*b*), and logarithm of the current density (log|*j*|). This plot is commonly used to evaluate catalytic performance and to identify the possible HER mechanism. A lower Tafel slope indicates that the electrocatalyst can achieve higher current densities at lower overpotentials, suggesting more efficient charge transfer kinetics and enhanced HER activity.

Cyclic voltammetry (CV) measurements are performed for a minimum of 30 cycles to assess the catalytic efficacy of the HER process. These measurements were executed at a scan rate of 5 mV s^−1^ within a potential range of 0 to −1 V (*vs.* the Reversible Hydrogen Electrode, RHE). The Tafel slope was measured from linear sweep voltammetry (LSV) experiments at a consistent scan rate of 5 mV s^−1^. Electrochemical Impedance Spectroscopy (EIS) measurements were also carried out to analyze the charge transfer behavior of the synthesized catalyst.^26^

## 3. Results and discussion

### 3.1. X-ray analysis

**Fig. 1:**
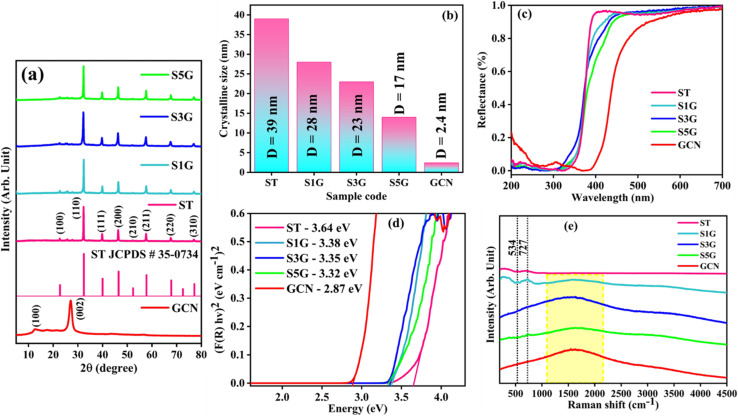
(a) X-ray diffraction plots, (b) calculated crystallite size using Scherrer relation, (c) UV-vis diffuse reflectance spectra, (d) bandgap obtained from the Kubelka–Munk relation fitting and (e) Raman spectra of the synthesized pure SrTiO_3_, g-C_3_N_4_ and SrTiO_3_/g-C_3_N_4_ composite samples.

The phase and crystal structure of pure SrTiO_3_, g-C_3_N_4_ and SrTiO_3_/g-C_3_N_4_ (1, 3 and 5% of g-C_3_N_4_) composites measured and analysed using a PANalytical Xpert-Pro instrument are shown in Fig. 1a. The tetragonal phase of SrTiO_3_ with space group *Pm*3*m* matches with JCPDS file no. 35-0734. The small peak observed at 25.3° belongs to the TiO_2_ anatase phase, originating from the precursor used to prepare SrTiO_3_.^22^ The XRD pattern of g-C_3_N_4_ shows two broad peaks at 2*θ* = 13.2° and 27.3° that are attributed to graphitic carbon nitride with crystallographic planes (100) and (002). The first peak is due to the in-plane alignment with a repeated periodicity between the triazine or heptazine, while the latter is ascribed to the planar graphitic interlayer periodicity of g-C_3_N_4_.^27^ Compared to the sharp diffraction peaks of SrTiO_3_, the peaks of g-C_3_N_4_ are broader and less sharp, which may be due to the smaller size and lower crystallinity of g-C_3_N_4_. No diffraction maxima of g-C_3_N_4_ were observed in the SrTiO_3_/g-C_3_N_4_ composite because of the low mass concentration; however, it is confirmed by Raman analysis and discussed in the upcoming sections. The average crystallite size estimated using Scherrer's relation was found to decrease systematically due to the incorporation of g-C_3_N_4_ with SrTiO_3_, as shown in Fig. 1b.

### 3.2. UV-vis DRS spectroscopic analysis

The optical characteristics of SrTiO_3_, g-C_3_N_4_ and the resultant composites with different mass ratios were assessed by UV-vis diffuse reflectance spectroscopy (DRS). The reflectance spectra of all the prepared samples show a decrease in reflectance after the incorporation of g-C_3_N_4_ in SrTiO_3_, indicating light absorption ability of the heterostructure samples.^28^ The bandgap was estimated using the Kubelka–Munk function, and it reveals the incorporation of g-C_3_N_4_ in SrTiO_3_ to decrease the bandgap of composites because of the interfacial heterojunction formation, electronic interactions, defect states, and synergistic optical properties of the composite materials, making it suitable for electrocatalytic applications.^29^

### 3.3. Raman spectra analysis

The Raman spectra of hydrothermally synthesized SrTiO_3_ with an observed band at 534 cm^−1^ are attributed to the first order Ti–O–Ti bending mode with very low intensity and their wavenumbers are assigned to the TO_4_ phonon, respectively.^30^ The 727 cm^−1^ band is due to second order Ti–O stretching modes in SrTiO_3_ that arise from the breaking of the crystal symmetry. The broad maxima in the 1000–2400 cm^−1^ region in the Raman spectra of g-C_3_N_4_ are due to the combination of structural and vibrational factors. Unlike pristine graphite, the incorporation of nitrogen creates structural defects and chemical heterogeneity, resulting in the inhomogeneous widening of vibrational modes.^31^ This broad maxima is a result of the overlap of several vibrational contributions, including the G-band (∼1570 cm^−1^), which is related to in-plane stretching of sp^2^-bonded carbon atoms, and the d-band (∼1350 cm^−1^), which is linked to structural disorder. In the S5G sample, the contribution of g-C_3_N_4_ is clearly seen with SrTiO_3_ (Fig. 1e), confirming the formation of composites.^32^

### 3.4. X-ray photoelectron spectroscopy

**Fig. 2:**
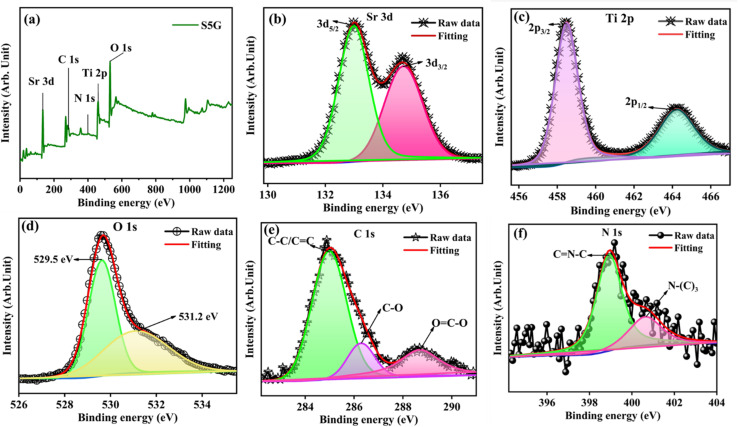
(a) XPS survey spectra of the S5G heterostructure sample, and the core level spectra of (b) Sr 3d, (c) Ti 2p, (d) O 1s, (e) C 1s and (f) N 1s.

X-ray photoelectron spectroscopy (XPS) for SrTiO_3_ with 5% of g-C_3_N_4_ (S5G) was performed. The XPS survey spectrum of S5G in Fig. 2a indicates the presence of constituent Sr, Ti, O, C and N elements. The Sr 3d peaks with binding energies of 132.9 eV and 134.72 eV with two deconvoluted curves, as shown in Fig. 2b, are ascribed to Sr 3d_5/2_ and Sr 3d_3/2_ levels, respectively.^11^ For Ti 2p, binding energies indexed at 458.5 eV and 464.2 eV are ascribed to the Ti 2p_3/2_ and Ti 2p_1/2_ levels, respectively, as shown in Fig. 2c. The peak at 458.5 eV is related to Ti^4+^ in the octahedron.^33^ The O 1s spectrum shows two peaks at 529.5 eV and 531.2 eV in Fig. 2d that are attributed to lattice oxygen (O_L_) and surface hydroxyl groups (OOH), respectively.^34^ The N 1s spectrum shows a peak at 389.9 eV corresponding to the pyridyl type linked to the sp^2^ hybridized nitrogen (C=N–C) in the heptazine ring, and the peak at 400.6 eV corresponds to the pyrrolic type related to the sp^3^ hybridized tertiary nitrogen (N–(C)_3_) (Fig. 2f). The C 1s spectrum in Fig. 2e exhibits peaks at 285 eV, 286.3 eV and 288 eV, ascribed to the band energies of C–C/C=C, C–O and O=C–O, respectively.^33^

### 3.5. Surface morphological and compositional studies

**Fig. 3:**
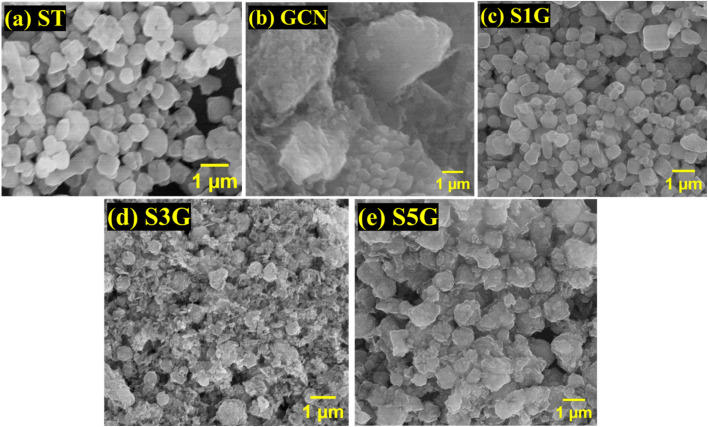
(a–e) SEM images showing the morphology of the ST, GCN, S1G, S3G, and S5G samples.

**Fig. 4:**
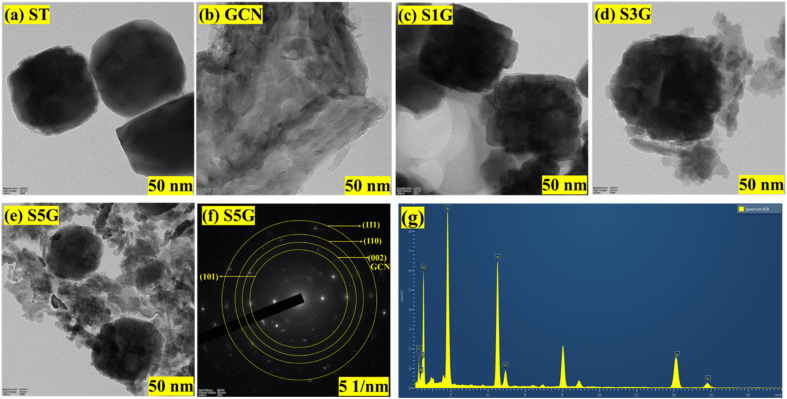
(a–e) TEM images of ST, GCN, S1G, S3G and S5G, and (f) SAED pattern and (g) TEM-EDX spectrum of the selected S5G composite.

The SEM image of SrTiO_3_ reveals the formation of a tetragonal structure in Fig. 3a. Fig. 3b indicates the successful formation of the graphitic stacking structure.^10,35^ The arrangement of SrTiO_3_ is evenly distributed across the g-C_3_N_4_ surface, as shown in Fig. 3c–e. High-resolution transmission electron microscopy (HR-TEM) images of SrTiO_3_, g-C_3_N_4_, SrTiO_3_ (with 1, 3, and 5% of g-C_3_N_4_) are presented in Fig. 4a–e. The TEM image of SrTiO_3_ reveals tetragonal morphology and g-C_3_N_4_ shows the formation of a stacked sheet-like layer, indicating the formation of a 2D structure. Fig. 4c–e vividly shows the formation of the composite, tetragonal SrTiO_3_, along with the layer of graphitic carbon nitride.^27,36^ The SAED image of the S5G composite in Fig. 4f shows the (002) plane of g-C_3_N_4_ and (111), (110) and (101) planes of SrTiO_3_, confirming the formation of the composite. The TEM-EDX spectrum of S5G in Fig. 4g shows the presence of Sr, Ti, O, C and N elements in the composites in the desired ratio.^37^

## 4. Electrochemical studies

Electrochemical measurements were executed in a three-electrode setup utilizing an OrigaMaster 5 workstation. The counter electrode is a platinum (Pt) wire, and a saturated silver/silver chloride (Ag/AgCl) electrode served as reference with a potential of approximately 0.197 V. The observed decrease in bandgap in the SrTiO_3_/g-C_3_N_4_ composites arises from the interfacial heterojunction formation, electronic interactions, defect states, and synergistic optical properties of the composite materials. As an acidic electrolyte, a 0.5 molar solution of sulfuric acid (H_2_SO_4_) was used. For the working electrode, the composite (SrTiO_3_/g-C_3_N_4_) was coated over a 5 × 5 cm size of carbon cloth. Before this coating, the carbon cloth was subjected to an intense cleaning procedure, comprising numerous washes with ethanol and sonication for 60 minutes.^25,38^ For making the catalyst slurry, 3 mg of the respective sample (ST, S1G, S3G, S5G, and GCN) was mixed with deionized water (700 µL), ethanol (200 µL), and Nafion (50 µL). This slurry was ultrasonicated for 60 minutes to attain a homogeneous ink. A 34.5 µL of the prepared ink was drop-casted onto one side of the carbon cloth, resulting in a working electrode with a surface area of 0.25 cm^2^. Finally, the carbon cloth cast with the SrTiO_3_/g-C_3_N_4_ ink was dried at ambient temperature for 24 hours. For the sake of comparison, a bare carbon cloth was used for all the electrochemical measurements.^26,32^

### 4.1. Linear sweep voltammetry

**Fig. 5:**
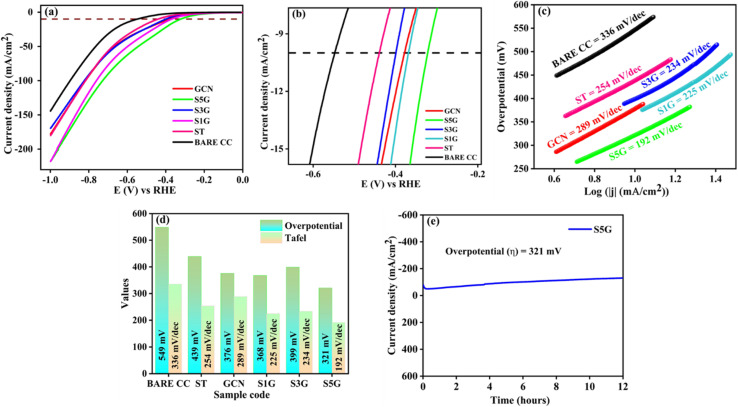
(a) Linear sweep voltammetry (LSV) curves, (b) zoomed-in plot of LSV curves at −10 mA cm^−2^, (c) Tafel plots, (d) comparison bar graph of overpotential and Tafel slopes and (e) chronoamperometric analysis of the optimal S5G composite catalyst.

Linear sweep voltammetry (LSV) curves were measured at a scan rate of 5 mV s^−1^ in order to assess the HER activity. The working electrode potential is linearly swept over time to measure the variation in current with applied potential in order to assess the catalytic activity of materials. The achieved overpotential values at 10 mA cm^−2^ for BARE CC, ST, GCN, S1G, S3G, and S5G were found to be 549, 439, 376, 368, 399, and 321 mV, respectively, as shown in Fig. 5a. In general, the low overpotential value for the S5G composite indicates that enhancing the composites ratio will lead to increased current density.^39^ In Fig. 5b, the zoomed-in plot of LSV at 10 mA cm^−2^ is shown.

### 4.2. Tafel analysis

Tafel curves were obtained from the LSV plot as depicted in Fig. 5c. The Tafel slopes of BARE CC, ST, GCN, S1G, S3G, and S5G were determined to be 336, 254, 289, 225, 234, and 192 mV dec^−1^, respectively. Among them, the S5G sample exhibited the lowest Tafel slope of 192 mV dec^−1^, indicating enhanced catalytic efficiency.^11^ A lower Tafel slope suggests that the catalyst can achieve higher current densities with lower overpotentials. Since catalysts with lower Tafel slopes require low additional energy to accelerate the reaction rate, they are considered more efficient in promoting the hydrogen evolution reaction (HER). The bar graph comparison for pure components and composites is depicted in Fig. 5d.

### 4.3. Chronoamperometric test

The stability of the SrTiO_3_ electrocatalyst with 5% g-C_3_N_4_ is explored by chronoamperometry test (CA) at relevant potential values using the LSV experiment for a duration of 12 h. Fig. 5e shows the stability of the S5G catalyst from the cathodic polarization curve scanned at a rate of 50 mV s^−1^.^40,41^ The curve indicates a slight increment from the initial current density over a 12 h period, indicating the enhanced stability of the heterostructure catalyst S5G in the acidic medium. Therefore, SrTiO_3_ with 5% g-C_3_N_4_ ensured that the composite can significantly elevate the electrochemical efficacy for the HER process in the acidic medium.

### 4.4. Electrochemical impedance spectroscopy

**Fig. 6:**
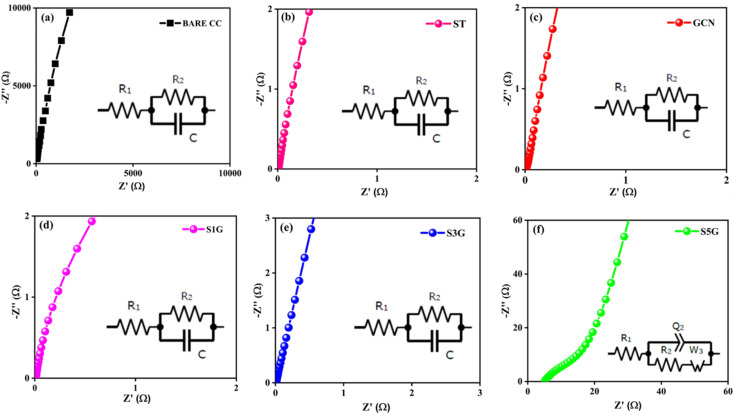
Electrochemical impedance spectroscopy (EIS) Nyquist plots of the (a) BARE CC, (b) ST, (c) GCN, (d) S1G, (e) S3G and (f) S5G samples, along with their respective equivalent circuits.

The Nyquist curves obtained from electrochemical impedance spectroscopy (EIS) yield details about the charge transfer kinetics at the interfaces. For pure ST, S1G, S3G and BARE CC (Fig. 6a–e), the equivalent circuits are also shown in the insets of Fig. 6, as derived from eqn (5),

5 *Z* = *R*_1_ + (*R*_2_/*C*)

where *R*_1_ represents the solution resistance, *R*_2_ denotes the charge-transfer resistance, and *C* corresponds to the double-layer capacitance. The EIS curve of S5G presented in Fig. 6f corresponds to the following equation (eqn (6)),

6 *Z* = *R*_1_ + *Q*_2_/(*R*_2_ + *W*_3_)

where *Q*_2_ is for the constant phase element, *R*_2_ is the charge-transfer resistance and *W*_3_ represents the Warburg diffusion element, indicating diffusion-related processes in the electrode.

EIS measurements were performed in the frequency ranging from 100 kHz to 1 Hz. Among all the samples, S5G exhibits the smallest semicircle in the Nyquist plot, indicating the lowest charge transfer resistance (*R*_ct_). This ensures that the S5G composite is optimal, as it shows a minimized *R*_ct_ value and more efficient charge transfer at the electrode–electrolyte interface.^26^

### 4.5. Long-term stability

**Fig. 7:**
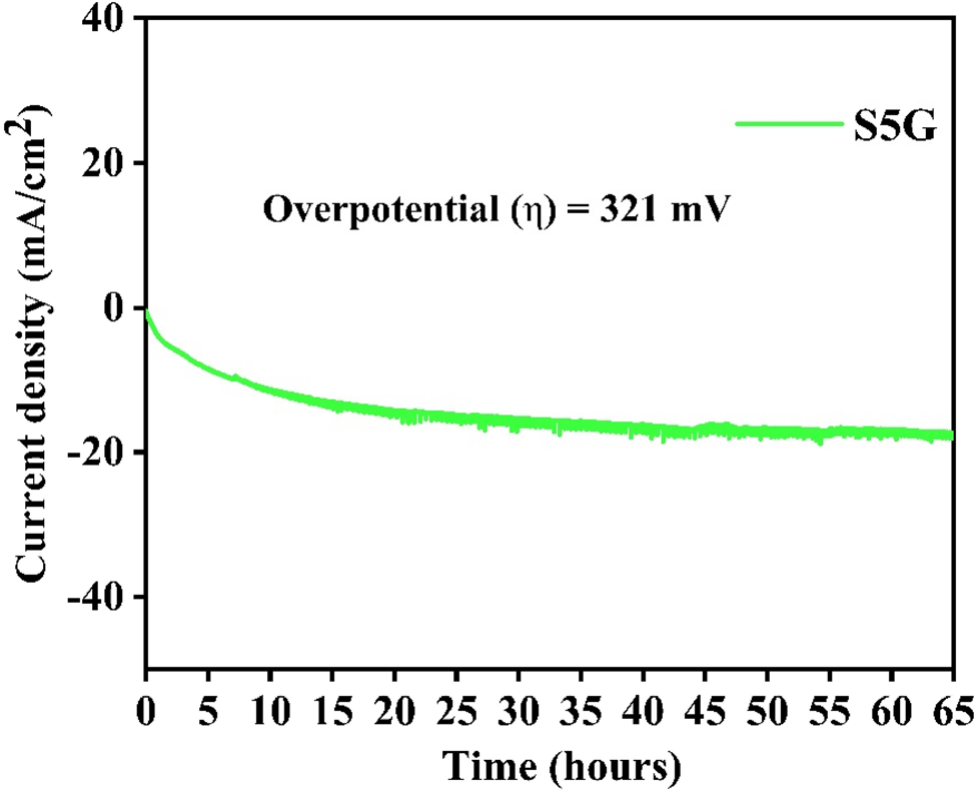
Chronoamperometric test of the optimal S5G composite catalyst.

The stability of the electrocatalyst SrTiO_3_ with 5% g-C_3_N_4_ is explored by a chronoamperometry test (CA) as shown in Fig. 7 at a relevant potential value from the LSV experiment for a duration of 65 h. The current density shows a decrease to around ∼10 mA cm^−2^ for the first few hours, which is attributed to the stabilization of active sites. Following this, the current density remains nearly constant throughout the testing time, maintaining a near constant current density at around ∼18 mA cm^−2^, indicating significant electrochemical durability for 65 h. The overpotential value of 321 mV ensures better HER activity. The sample S5G possesses good stability for possible long-term hydrogen evolution applications.

## 5. Conclusion

The SrTiO_3_/g-C_3_N_4_ heterostructure sample was optimized to boost the hydrogen evolution reaction (HER) activity. Electrochemical analysis for HER performance in an acidic environment demonstrated improved efficiency, with the heterostructure composite S5G achieving a low overpotential of 321 mV, Tafel slope of 192 mV dec^−1^ and a significant stability of 65 h. The integration of g-C_3_N_4_ with SrTiO_3_ significantly improves HER activity compared to the individual pure SrTiO_3_ and g-C_3_N_4_ components, making it a viable material for sustainable and efficient hydrogen generation. The synergy between SrTiO_3_ and g-C_3_N_4_ in the heterostructure sample enhances the electrochemical active surface area, leading to more efficient charge transfer for effective hydrogen production. Overall, the composite comprising SrTiO_3_ with 5% g-C_3_N_4_ stands out as a promising candidate for the development of sustainable and high-performance HER catalysts. The hetero-structured SrTiO_3_ and g-C_3_N_4_ system also has scope for light-assisted catalytic performance, which may enable efficient transfer of photo-excited electrons between interfaces, which may result in charge separation and provide additional electrons for the hydrogen evolution reaction. The combination of light irradiation and electric bias application through photo-assisted electrocatalysis may boost hydrogen evolution activity by raising the charge carrier density and enabling interfacial charge transfer. This strategy has the potential to gain practical value because the combination of light and electricity would create a process that operates closer to solar-assisted hydrogen production.

## Conflicts of interest

There are no conflicts to declare.

## Data Availability

The datasets generated during and/or analysed during the current study are not publicly available due to institutional policy decision but are available from the authors on reasonable request.
